# Pupillary response to real, illusory, and implied motion

**DOI:** 10.1371/journal.pone.0254105

**Published:** 2021-07-01

**Authors:** Serena Castellotti, Carlo Francisci, Maria Michela Del Viva

**Affiliations:** Department of Neurofarba, University of Florence, Florence, Italy; Tokai University, JAPAN

## Abstract

The perception of moving objects (real motion) is a critical function for interacting with a dynamic environment. Motion perception can be also induced by particular structural features of static images (illusory motion) or by photographic images of subjects in motion (implied motion, IM). Many cortical areas are involved in motion processing, particularly the medial temporal cortical area (MT), dedicated to the processing of real, illusory, and implied motion. Recently, there has been a growing interest in the influence of high-level visual processes on pupillary responses. However, just a few studies have measured the effect of motion processing on the pupil, and not always with consistent results. Here we systematically investigate the effects of real, illusory, and implied motion on the pupil diameter for the first time, by showing different types of stimuli (movies, illusions, and photos) with the same average luminance to the same observers. We find different pupillary responses depending on the nature of motion. Real motion elicits a larger pupillary dilation than IM, which in turn induces more dilation than control photos representing static subjects (No-IM). The pupil response is sensitive even to the strength of IM, as photos with *enhanced* IM (blur, motion streaks, speed lines) induce larger dilation than simple *freezed* IM (subjects captured in the instant they are moving). Also, the subject represented in the stimulus matters: human figures are interpreted as more dynamic and induce larger dilation than objects/animals. Interestingly, illusory motion induces much less dilation than all the other motion categories, despite being seen as moving. Overall, pupil responses depend on the individual perception of dynamicity, confirming that the pupil is modulated by the subjective interpretation of complex stimuli. We argue that the different pupillary responses to real, illusory, and implied motion reflect the top-down modulations of different cortical areas involved in their processing.

## Introduction

Motion perception, defined as the perception of change of position of an object over time, is a crucial function for successfully interacting with a dynamic environment and is fundamental for survival [1, 2]. The neural activity of the medial temporal/medial superior temporal areas (MT+/V5+ and MST) of monkeys is dedicated to process directionality and speed [3–7] of moving visual stimuli. This seems to be true for humans as well, as demonstrated by anatomical [8–10], PET [11–13], and fMRI studies [14–17]. Damages to the MT area in humans may selectively impair movement perception [18].

Humans can perceive motion even in static images due to the combined effect of particular structural features, such as colors, contrast, shapes, and their position (illusory motion) [19–22]. Visual motion areas dedicated to real motion processing have been shown to be active in the presence of illusory motion as well [23–25]. For instance, apparent motion of figures defined by illusory contours evokes a considerable fMRI activation in V2 and MT/MST [23]. PET studies found an increase of regional cerebral blood flow (rCBF) in V5 while observers perceived illusory motion, similar to that recorded while the same observers viewed a physically moving stimulus [25]. Surprisingly though, illusory motion stimuli do not elicit significant activity in V1, which is highly active when real motion stimuli are presented. Cortical areas other than the visual cortex are also activated during observation of motion illusions, but not during the presentation of real moving objects [25]. It has been speculated that the activity in these non-visual areas, particularly the cingulate gyrus, is related to attention [25]. Overall, perception of illusory motion seems to depend on the activity of specialized visual motion areas but also on the activity of other brain regions that are not involved in the processing of real motion [25].

Visual motion can be also perceived in stimuli that are static in nature but representing motion situations (*Implied* motion, IM). That is, we are able to perceive a dynamic visual scene from static images that show directional information or particular form elements [26–33]. The impression of movement of a subject in a static image can be conveyed by different cues, such as action sequencing, action freezing, motion streaks/speed lines, or motion blur in the direction of the represented movement [34–41]. The dynamic state of a subject in a still image can even be inferred from the complex shape of the subject itself, as its postural expression, arms or legs position, or muscular tension [32, 37, 42], showing that the extraction of motion information from stationary pictures is a process of inference, that can also involve prior experiences [28].

Several fMRI and neurophysiological studies suggested that the perception of real and implied motion share the same neural substrates. It has been shown that the same subpopulations of cells in dorsal areas responding to real motion are also responsive for implied motion, showing that they are indifferent to the cue (implied or real) that generates the motion [43, 44]. Even motion after-effect (MAE), caused by prior exposure to motion in the opposite direction [45] and mediated by MT [14], can be induced by viewing a series of static photographs with implied motion in a particular direction [32]. Furthermore, the prototypical visual motion areas (MT+/MST) seem to be more active with real-life pictures depicting implied motion rather than static subjects [26, 28, 29, 31, 44]. For example, stronger fMRI activation within MT+/MST areas has been found with photographs of athletes in action compared with humans at rest, or even with nature scenes depicting inanimate motion [44]. MT activity resulted to be modulated in relation to the degrees of dynamism and speed inherent within images [29, 31]. Even the observation of a sculpture specifically settled out to convey an impression of dynamicity, was found to influence intracortical excitability of the motor cortex [26]. Several other studies found that V5 is involved in extracting motion information from complex pictorial representations, such as figurative paintings depicting subjects in movement [46–48] or even abstract paintings without any representational content [47, 49]. Given the existing evidence for specialized cortical areas for visual processing of the human body (Extrastriate Body Area, EBA) and faces (Fusiform Face Area, FFA, and Occipital Face Area, OFA) [50–57], the investigation of motion’s processing should take into account the nature of the subject. Indeed, the perception of implied motion operates differently when the subject presented is a human figure, due to our inherent tendency to infer other people’s intentions from their actions [58–60]. Several studies showed higher activation of motion areas for stimuli depicting humans in action than inanimate objects depicted as moving [61]. Besides MT+, human-implied motion seems also to involve activation of a larger number of cortical areas, than non-human IM, including the superior temporal sulcus, the motor cortex, and the mirror system areas [29, 62–64].

Recently, there has been a growing interest in high-level cognitive processes, elaborated at cortical level, that have an influence on the pupillary response. Indeed, it is now well established that the pupillary light reflex (PLR), the low-level mechanism that simply regulates the amount of light entering the eye [65, 66], is not the only factor that modulates the pupil size. Pupil size can also dilate under steady lighting conditions in response to a range of cognitively relevant factors including changes in stimulus salience, mental effort, and internal states [67–70]; all linked to an increased level of arousal mediated by the activation of the sympathetic nervous system [67, 71–73]. More recently, pupillometry studies have focused on the investigation of the effects of high-level visual processes on the pupil diameter, such as visual attention [74–80], perceptual illusions [81–83], visual imagery [84, 85], high-level processing of images content [74, 75, 86, 87], and interpretation of complex stimuli like paintings [88].

Despite several studies on top-down modulations of the pupil, the effect of cognitive interpretation of motion has not yet been systematically investigated. Very few studies tried to measure the pupil size in response to motion processing; and those who did provided contrasting results [89, 90]. As an example, the onset of coherent movement generated in a pattern of random dots has been found to trigger pupil constriction, that cannot be accounted for in terms of PLR [90]. This effect has been linked to the changes in neural activity in the visual cortex occurring in case of sudden changes of stimulus properties [90–92]. Instead, pupil dilation was found in response to illusory motion in peripheral drift images and this result was attributed to the physiologically arousing nature of illusory motion [89].

These findings suggest that cortical mechanisms involved in motion processing may influence pupil size independently on luminance changes, but they do not allow to reach exhaustive conclusions on how the pupil responds to motion, especially in connection to the different types of motion. These studies perhaps suggest the existence of different pathways and mechanisms regulating pupil dilation in response to real and illusory motion but given the differences in stimuli and procedures, a comparison of them may be not appropriate.

In the present work, we systematically investigate the effect of motion perception and interpretation on the pupillary response, by showing different types of stimuli with the same average luminance to the same group of observers. To explore the effects of real, illusory, and implied motion we use respectively movies with moving objects/animals or humans, visual illusions that convey the perception of motion, and photographs with dynamic scenes of moving objects/animals or humans. Since human images, both static and in motion (e.g., biological motion) are processed by selective neural processes, we choose stimuli both with humans and animals or objects to explore the possibility that they might have different effects on the pupillary response, a question never experimentally investigated so far.

It is known that pupil responses are modulated by the subjective interpretation of images [81, 84, 87, 88]. For this reason, we assess for each image the degree of motion perceived by participants with a rating test. The results of the test are used in the analysis of pupil responses for each motion category. Using a single experimental paradigm and stimuli with matching physical luminance, our study may allow a comparison of the effect of different types of motion on the pupil size, broadening the knowledge about the influence of motion cognitive processing on the pupil.

## Materials and methods

### Participants

Twenty-four participants (15 women and 9 men, mean age = 25 ± 3 years) took part in the experiment. All selected participants had a normal or corrected-to-normal vision (by contact lenses) and did not take any type of medication. Participants were asked to not wear eye makeup. Participants were unaware of the aim of the experiment and gave written informed consent prior the participation. All experimental procedures were approved by the local ethics committee (Comitato Etico Pediatrico Regionale—Azienda Ospedaliero-Universitaria Meyer—Firenze FI) and were compliant with the Declaration of Helsinki.

### Apparatus and set-up

Stimuli were displayed on a 51 x 29 cm ASUS monitor (MX239H) with a resolution of 1920 x 1080 pixels. The observer was positioned at a 57 cm distance from the monitor with a chin rest to stabilize the head position. Stimulus presentation and data collection programs were developed using the Psychophysics Toolbox extensions [93–95] for Matlab (R2016b version). The experimental procedure was carried out in a dark room with no illumination other than the display screen. The diameter of both eyes was measured with a CRS LiveTrack FM system (Cambridge Research Systems) at 60 Hz, collecting 240 data points per trial. Throughout the whole pupil recording, the experimenter monitored the corrected position of the participants’ eyes by using a camera (using QuickTime software) on a dedicated computer (iMac Retina 5K, 27-inch, mid 2015 3.3 GHz Intel Core i5 processor, MacOs Sierra software 10.12.6). Participants’ manual responses to the final rating test were provided on a standard Dell keyboard. JASP software (Version 0.8.6) was used for statistical analyses.

### Procedure

**Fig 1:**
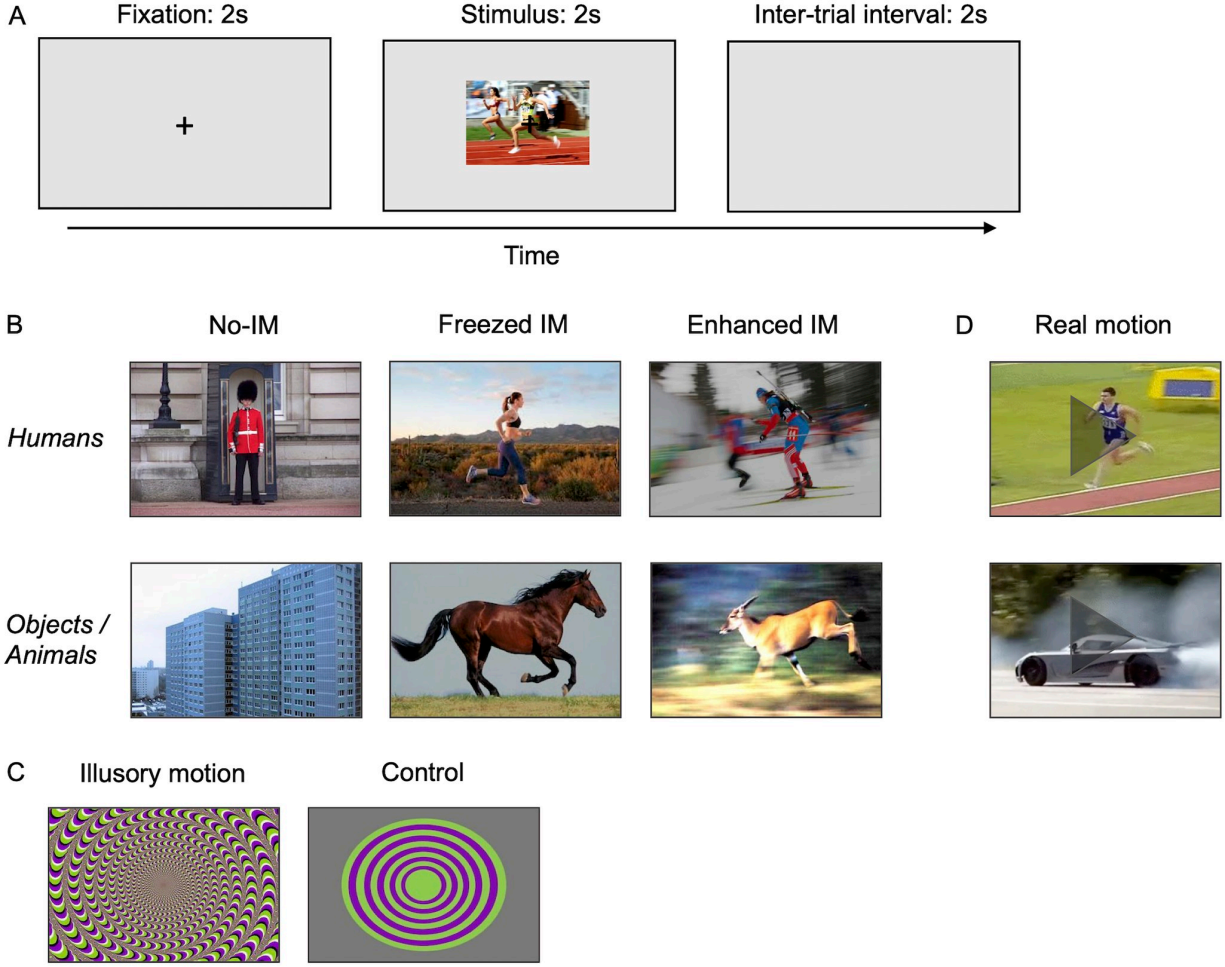
Procedure and stimuli. **(A)** Schematic of the experimental paradigm. Image shown is n° 67 of S1 List. **(B)** Examples of photos representing different motion categories and content. Images (from left to right) are n° 44, 59, 63, 4, 18, and 22 of S1 List. **(C)** Example of motion illusion (n° 88 of S1 List) and its control image. **(D)** Examples of movies of animated and inanimate subjects, represented here with a single frame. Images (from up to down) are extracted from movies n° 76 and 35 of S1 List. Images shown are copyright-free. All experimental stimuli, except controls for illusions, were retrieved from Internet (see S1 List for web sources).

The experimental procedure consisted of 100 trials, divided into six blocks: from blocks 1 to 4 static stimuli (photos and illusions, 70 total) were shown, block 5 consisted of 20 movies, and in block 6 10 illusions’ control images were presented. The sequence of stimuli presentation was randomly predetermined and kept the same for all observers. Before the first and the third block, a standard nine-point calibration routine was run to ensure the correct working of the eye tracker. There was a pause of about 5 minutes between blocks. Trials started with the presentation of a fixation slide (124 cd/m2) for 2 seconds, with a black fixation cross (5 x 5 mm) in the center. This was followed by the presentation of one stimulus for 2 seconds, keeping the fixation cross visible in the center of the screen. Participants were required to look at the stimuli without performing any other task, and they were not aware of the motion rating test we proposed after the pupil recording. To avoid any effect of eye movements on pupil size, during fixation and stimulus presentation participants were instructed to refrain from blinking and to maintain their gaze on the fixation cross without exploring the stimuli with eye movements. Fixation was monitored by the experimenter (see Apparatus and set-up section). The luminance of the background screen was kept constant at 124 cd/m. Each trial was followed by the presentation of a blank screen (124 cd/m2) of 2 seconds, while pupil recording was suspended. During this inter-trial interval, participants were allowed to blink and rest their eyes before the next trial, preventing the build-up of after-images and allowing the pupil to return its baseline size (Fig 1A).

After the recordings, all the static stimuli (photos and illusions) were presented again in the same sequence to the observers without time limitation. Observers were asked to rate from 0 to 2 the degree of motion (implied or illusory) they perceived in each (0—No motion; 1—Weak motion, 2—Strong motion), by pressing a computer key. The results of the rating were used as detailed in the Data processing section.

The complete procedure took about 45 minutes per observer, about 30 minutes of which were for pupil recordings.

### Stimuli

Before the experiment, an initial set of 120 photos and illusions had been rated by a group of 15 naive judges that did not take part to data collection, on a 3 levels Likert scale—0 (no motion), 1 (weak motion), 2 (strong motion). The images that reached the higher agreement between the judges (never less than 70%) were selected in the final sample of stimuli. Each image was then nominally assigned to one of the different categories of the experiment, based on this motion rating.

The selected images were 60 realistic photos and 10 optical illusions (see S1 List for web sources). Photos consisted of 20 pictures of static subjects (nominal categorization as “No-implied motion”, No-IM), 40 pictures of dynamic subjects (“Implied motion”, IM). Twenty of these photos capture subjects while they are moving (“*Freezed* IM”), and 20 were purposely created by photographers to enhance the presence of motion—for example showing action sequencing, motion streaks, speed lines, blurred subjects, or blurred backgrounds (“*Enhanced* IM”). These three photos’ categories included 10 images of objects or animals and 10 images of human figures each. Examples of photos are reported in Fig 1B. Photos of static subjects (objects, animals, or humans) were used as controls for implied motion stimuli (*freezed* and *enhanced* IM) because they are realistic full-color images with a complex content without containing any embodied motion information. The set of optical illusions (“Illusory motion”) contained 10 illusions that were rated in the initial test as those conveying the strongest impression of motion (example in Fig 1C).

As a control for illusions, we created 10 matching images, sharing the same patterns of color saturation and luminance, without conveying the impression of illusory motion (“Illusions’ control”) (example in Fig 1C). That is, they consist of basic geometric shapes (squares, rectangles, and circles) on a grey background, with repeating symmetric patterns that disrupt the illusion of motion [19, 89].

To compare the response to implied motion in static pictures with real motion, we also used a set of 20 movies (“Real motion”), also divided into 10 videos of moving objects or animals, and 10 videos of moving people (see S1 List for web sources). All movies were cut to 2-seconds clips of 120 frames (examples in Fig 1D).

All images and movies were rescaled to a common resolution (28.35 pixels/cm), and they were resized (conserving proportions) to either a width or a height of 340 pixels, with the other side ranging from 171 to 340 pixels. By looking at such small images, observers could have a gist of the whole image without having to explore its parts with eye movements [88, 96].

Finally, the luminance of all images and movies (frame by frame) was modified and rescaled to the value corresponding to the average luminance of the whole set (25 cd/m2), to obtain our experimental stimuli. The Fourier power spectra of our stimuli have the same general shape and the majority of the power lie outside the 2–10 c/deg frequency range; this may dilute a possible constriction exerted by these frequencies [97, 98].

### Data processing

Raw data recorded by the eye-tracker consisted of measurements of the width and the height of both pupils (in pixels). Right and left pupil diameters were averaged, and the resulting value was converted from pixels to millimeters, based on the instrument’s recording of a 4 mm artificial pupil, positioned at the location of the observer’s left eye.

The analysis of the pupil responses elicited by different categories of stimuli follows a method widely used in literature for this type of experiments [86, 88, 96]. For each observer, a baseline pupil diameter was calculated by averaging pupil diameter recorded over the last 500 ms of the fixation slide in each trial. This baseline was subtracted from each recording of that observer over the whole 4 sec period [88, 99]. Baseline-corrected pupil size as a function of time of each observer was calculated by averaging the pupil traces of the ten stimuli of each stimulus category. Final traces for each category were then obtained by averaging all observers’ traces. The slopes of the traces were evaluated by fitting data in the interval between 400 ms and 650 ms from stimulus onset with straight lines. The slope values were compared by using *z*-tests.

An average pupil size was calculated for each category as follows. First the average pupil size of each observer for each image/video was calculated by averaging the baseline-corrected pupil values during the stimulus presentation (120 data points). Then, for each observer, these values were averaged over all ten stimuli of the same category. Finally, the averages for each category were obtained by averaging over all observers. Differences between the means of categories were assessed with ANOVAs, and pairwise comparisons were done with post-hoc t-tests (Bonferroni corrected). The size effect of differences between categories was evaluated by Cohen’s *d* statistics [100, 101].

The eye-tracker also recorded the observer’s eye position along the horizontal and vertical axes. To control fixation two different analyses were done. First, for all categories, we measured the average distance of the eyes, with respect to fixation, of all subjects during stimulus presentation in all trials. ANOVA was used for comparisons between average distances of categories. To control eye positions during the presentation of the stimuli, we measured root mean square errors (RMSE) of horizontal eye positions for all categories and we compared their distributions with Kolmogorov-Smirnov tests (KS).

Following the idea that the pupil size reflects the individual interpretation of the stimulus [81, 84, 87, 88], data were corrected based on the amount of motion perceived in each image by each observer participating to the experiment, assessed with the rating test. Pupillary responses to stimuli that participants did not categorize in agreement with the nominal classification were considered as "misinterpretations" and were excluded from the analysis. For instance, images assigned to the “Implied motion” nominal category in which, however, observers did not perceive motion (answer “0 –No Motion” in the test) were excluded from the mean of that category for those observers. In the same way, images belonging to the “No-implied motion” nominal category, for which participants perceived motion (answer “1—Weak motion” or “2—Strong motion”), were excluded from the mean of No-IM category as well. Percentage of misinterpretations across categories were compared with the Chi-square test (χ^2^). Pupil responses of observers which interpreted the same image differently were compared by using a non-parametric, one-tailed, Mann-Whitney ranking test. In those cases where only one misinterpretation occurred, the p-value was directly determined as the ratio of the rank of the outlier and the total number of observers. To assess the overall significance for the presence of the effect of individual motion interpretation on pupillary response, individual *p*-values were combined according to Fisher’s method [102].

## Results

**Fig 2:**
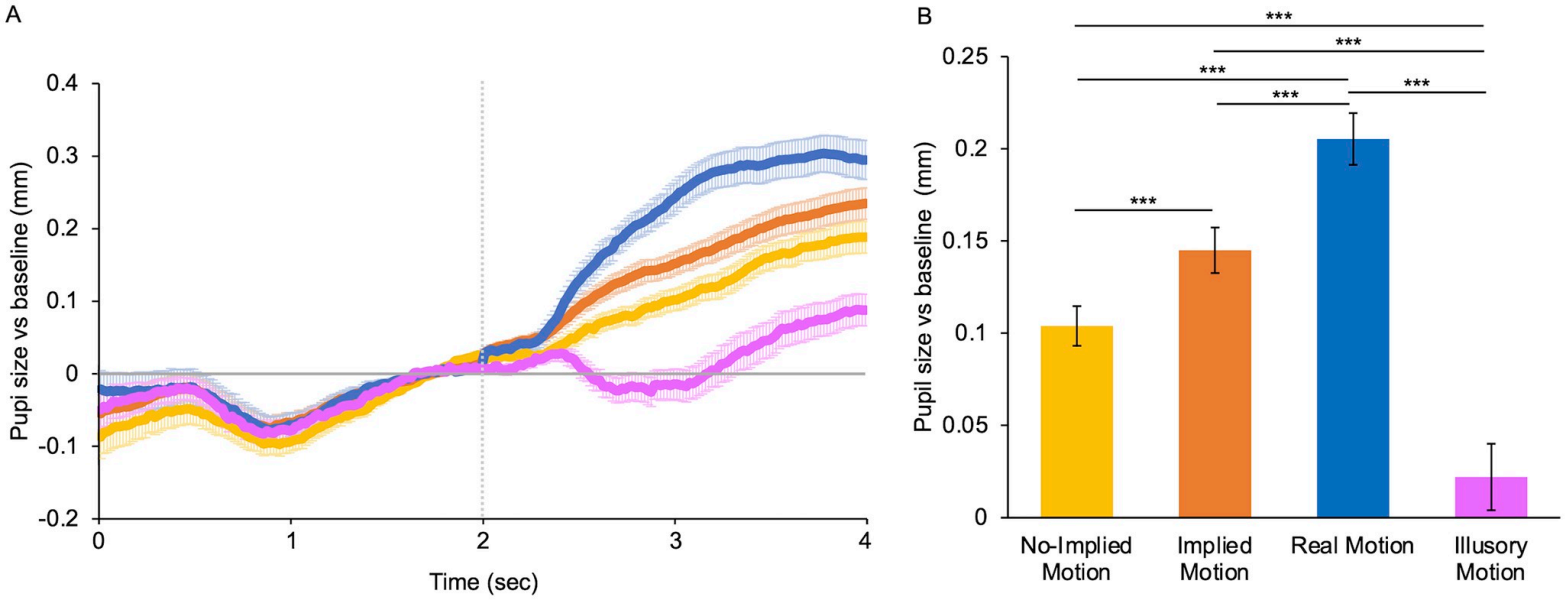
Mean pupillary response for different types of motion. **(A)** Baseline-corrected pupil size for different motion categories plotted as a function of time from trial onset. The horizontal line represents the baseline: data over the zero-line represent pupil dilation whereas data under the line represent pupil constriction. The vertical line indicates the stimulus onset. Error bars are SE. **(B)** Mean pupillary diameter for different categories of motion during stimulus presentation. Categories include both objects/animals’ and humans’ subjects. Yellow: photos with no implied motion (No-IM); orange: photos with implied motion (*freezed* and *enhanced* IM); blue: movies with moving subjects (real motion); purple: motion illusions (Illusory motion). Error bars are SE. Asterisks mark statistically significant pairwise comparisons across image categories: *** *p* < 0.001.

The main result of this work is reported in Fig 2. It clearly shows that real, implied, and illusory motion produce different pupillary responses, both in time course and magnitude.

Pupil dilation for the different motion categories during the first ~350 ms is almost the same, as expected with PLR in response to stimuli with the same mean luminance presented on a much lighter background (Fig 2A). However, if responses were based on stimulus’ luminance only, we would expect the same dilation and with the same time course for all types of motion stimuli. Instead, after about half a second, pupil responses begin to differ between motion categories. The slopes of the curves, where they start to differ (between 400 and 650 ms of stimulus presentation) are very different across conditions (pairwise z-test comparisons, *p* < 0.0001). The slope of the real motion curve is positive (0.005 ± 0.0002) and greater than that of the IM (0.003 ± 0.00007), that in turn is greater than that of No-IM (0.002 ± 0.0001), indicating the stronger the perception of movement, the faster the change in pupil size. After 650 ms from stimulus onset, pupil response to photos, both with IM (orange line) and without IM (yellow line), produce a similar path of progressive increase in pupil diameter, even though, on average, photos with IM caused more dilation. Instead, the initial large increase in dilation caused by movies (blue line) stops after just about a second of exposure, leading to a mostly stable pupil diameter over the last 800 ms. This stabilization can be understood by considering that +0.3 mm with respect to baseline corresponds to a pupil diameter of about 8 mm, which is the physiological limit for dilation [103].

Interestingly, the pupil response to motion illusions (purple line) turns out to be very different from that of photos and movies. Pupil size instead of increasing starts decreasing progressively, with a negative slope (-0.0034 ± 0.0001). After reaching constriction pupils remain constricted for about 500 ms. Finally, during the last second of recording, the pupil diameter increases progressively, however remaining much less dilated than for the other stimuli.

The average response to the different types of motion is reported in Fig 2B. Significant differences between the means of all categories are evidenced by ANOVA (*F*(3) = 64.64, *p* < 0.001). IM causes pupillary dilation (*M* = 0.14 ± 0.01 mm), statistically larger (*t*(3) = 4.74, *p* < 0.001) than that produced by No-IM (*M* = 0.10 ± 0.01 mm), with a *large* effect size (*d* = 0.9). These results suggest different effects on pupil size depending on whether a subject is perceived as dynamic or static.

Real motion causes the largest dilation (*M* = 0.21 ± 0.01 mm), statistically larger than that produced by IM (*t*(3) = -4.98, *p* < 0.001) and No-IM (*t*(3) = -7.72, *p* < 0.001), and the magnitude of the effects is *large* (*d* = 1.01) and *very large* respectively (*d* = 1.5). The average dilation induced by illusory motion is very small (*M* = 0.02 ± 0.02), almost absent, as expected from inspection of pupil traces, and significantly lower than that that produced by No-IM (*t*(3) = -9.12, *p* < 0.001), IM (*t*(3) = -9.12, *p* < 0.001) and real motion (*t*(3) = -10.33, *p* < 0.001), with *very large* to *huge* effect sizes (in order: *d* = 1.8; *d* = 1.15; *d* = 2.11).

**Fig 3:**
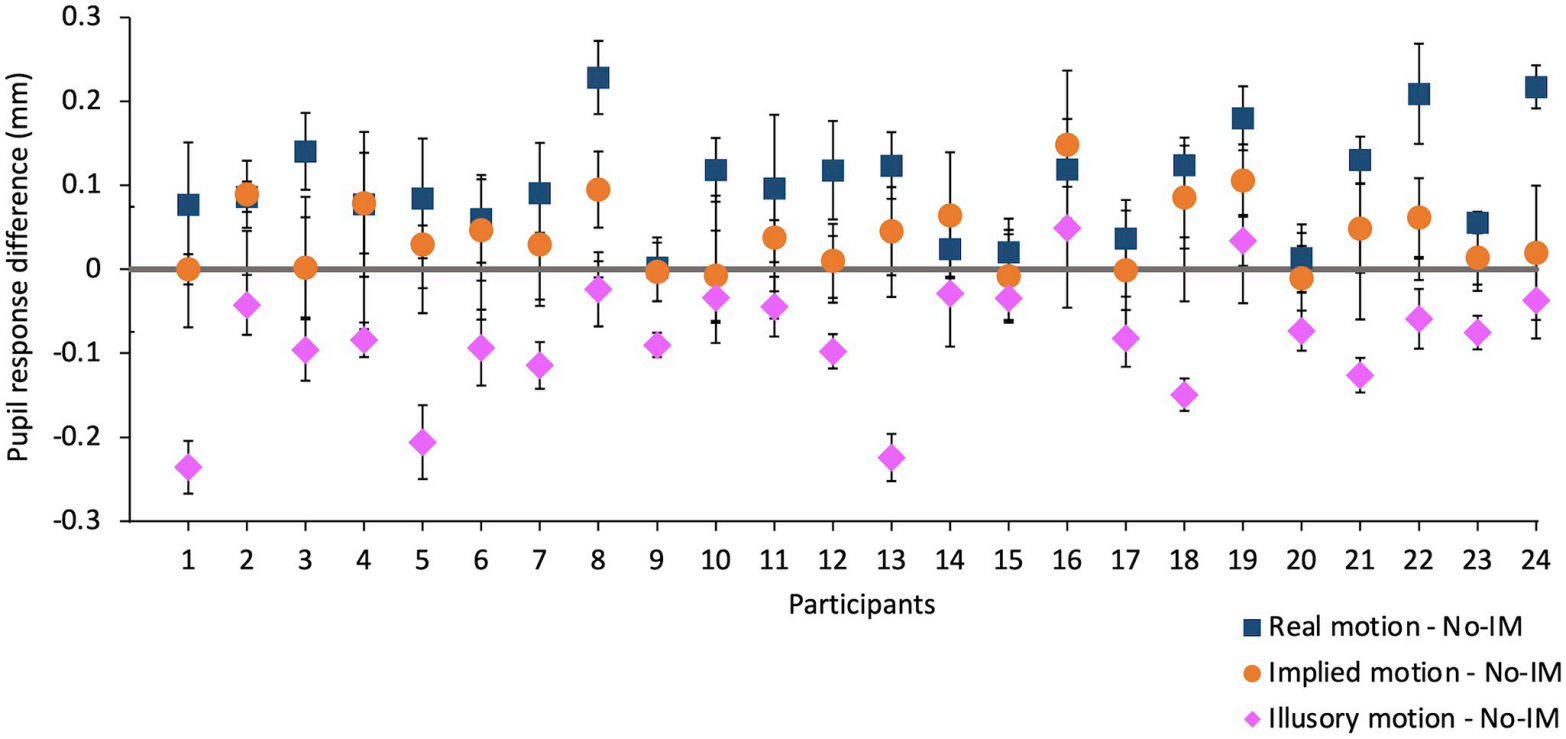
Individual differences between responses to no-implied motion vs real, implied, and illusory motion. Blue squares: differences between average pupil response to movies and photos with No-IM; orange circles: differences between average pupil response to photos with IM and photos with No-IM; purple rhombuses: differences between average pupil response to motion illusions and photos with No-IM. Error bars are the errors on the differences.

Individual data highlight the same differences between responses to real, implied, and illusory motion, as shown in Fig 3, where the differences in pupil response to photos of static subjects (No-IM), photos of dynamic subjects (IM), movies (real motion) and motion illusions (illusory motion) are plotted for each participant. The difference between pupil response to No-IM and real motion is positive for all observers; the difference between No-IM and IM is almost always positive (even if lower than that with real motion and negligible for some participants); whereas the difference between No-IM and illusory motion is negative for all observers, confirming group results.

It is known that motion viewing can trigger eye movements—for example to track a moving subject [104, 105], and that eye movements can influence pupil size [106]. For this reason, although our observers were instructed to keep fixation and their eye movements were monitored by the experimenter during recording, we measured the average position of their eyes with respect to the fixation cross, for the different stimulus categories. The average distance from fixation is minimal (No-IM: 2.90 ± 0.5 mm; IM: 1.90 ± 0.3 mm; Real motion: 2.07 ± 0.4 mm; Illusory motion: 1.80 ± 0.3 mm) and the same for all categories (ANOVA, *F*(3) = 1.88, *p* = 0.13). We also tested that the variation of eye positions along the horizontal axis did not differ between categories, by comparing the distributions of root mean square errors (RMSE). All pairwise comparisons do not highlight any differences between categories (all KS test yield *p* > 0.05).

**Fig 4:**
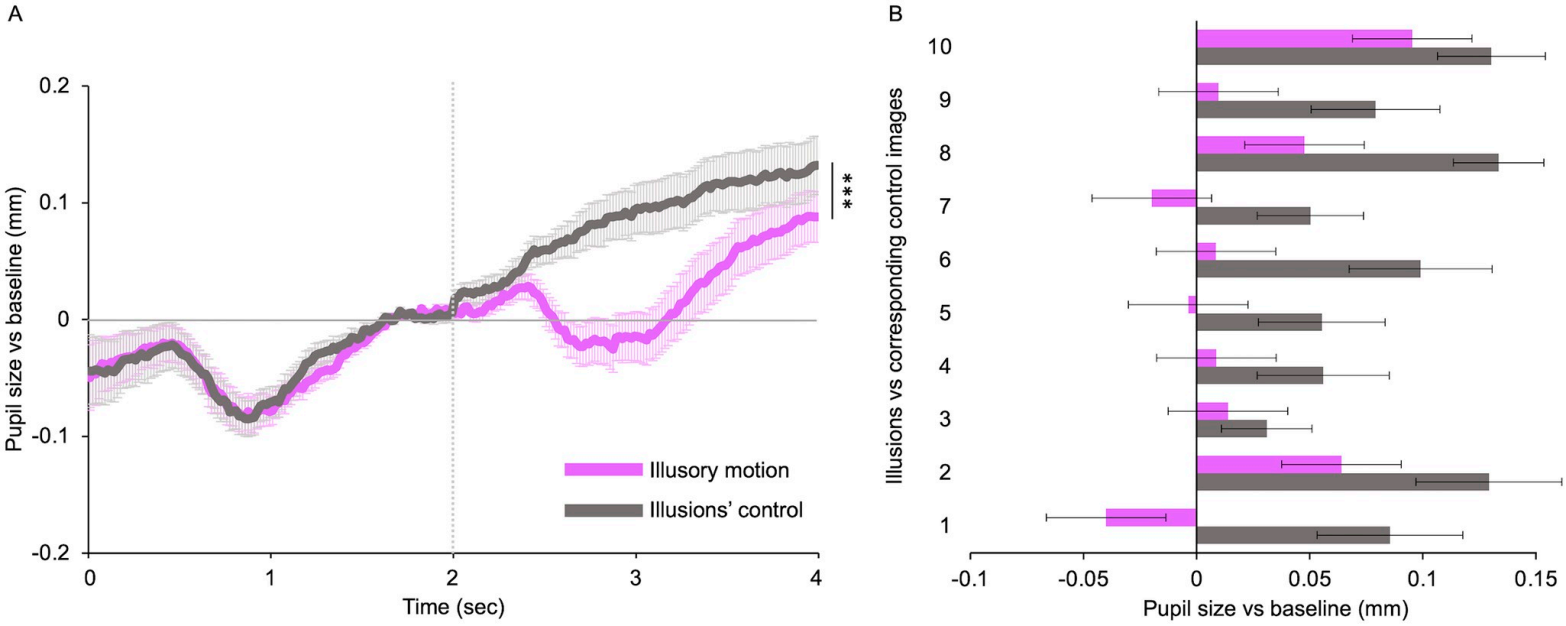
Control for illusory motion effect. **(A)** Baseline-corrected pupil size for illusory motion and illusions’ control, plotted as a function of time from trial onset. The horizontal line represents the baseline: data over the zero-line represent pupil dilation whereas data under the line represent pupil constriction. The vertical line indicates the stimulus onset. Error bars are SE. Asterisks mark statistically significant comparison across image categories: *** *p* < 0.001. **(B)** Mean pupillary diameter for each illusion and the corresponding control image. Purple: motion illusions; grey: illusions’ control images. Error bars are SE.

The peculiar effect on the pupil produced by illusory motion may be due to the different structural characteristics of the illusions compared with those of photos or movies. Indeed, while motion perception in movies is induced by real changes of objects’ positions during time, in photos this is achieved by representing dynamic scenes with subjects in action. Instead, the perception of visual motion in the illusions is due to specific images’ features such as the colors’ combination and the repeating asymmetric patterns arrangement [95, 99]. To assess the influence of these low-level visual properties on the pupil constriction observed for illusions, we compared responses to illusions with their control images (same color saturation, same shapes, same luminance, without conveying motion perception—see Fig 1C for examples) shown in Fig 4. The two categories induce very different effects. Control images induce pupillary dilation that slowly increases over time, while illusions cause a decrease followed by an increase of pupil size, as discussed before (Fig 4A). Paired sample t-tests show that the mean pupil dilation induced by the controls (*M* = 0.08 ± 0.02 mm) is statistically greater (*t*(23) = 5.23, *p* < 0.001) than that elicited by illusory motion (*M* = 0.02 ± 0.02 mm), with *large* effect size (*d* = 1.06). Individual illusions induce different effects on pupil size as shown in Fig 4B (purple bars). However, each of them elicits a pupil size smaller than that of its control image (Fig 4B, grey bars).

As discussed above (Fig 2), pupil dilation increases with the strength of the motion represented in the figurative stimuli, from photos with static subjects to photos with implied motion to real movies. It is then interesting to further investigate the effect of the strength of the motion represented in IM pictures. For this purpose, we re-analyzed our data by considering the type of IM and looking for possible differences between responses elicited by photos with motion blur or motion strikes (*Enhanced* IM), and those elicited by photos showing simple *freezed* IM (see Fig 1B for examples). Furthermore, given that, for social reasons, we have dedicated systems for the analysis of human motion, it is also interesting to re-analyze our data by considering the subjects represented in the stimuli: objects/animals versus human figures.

**Fig 5:**
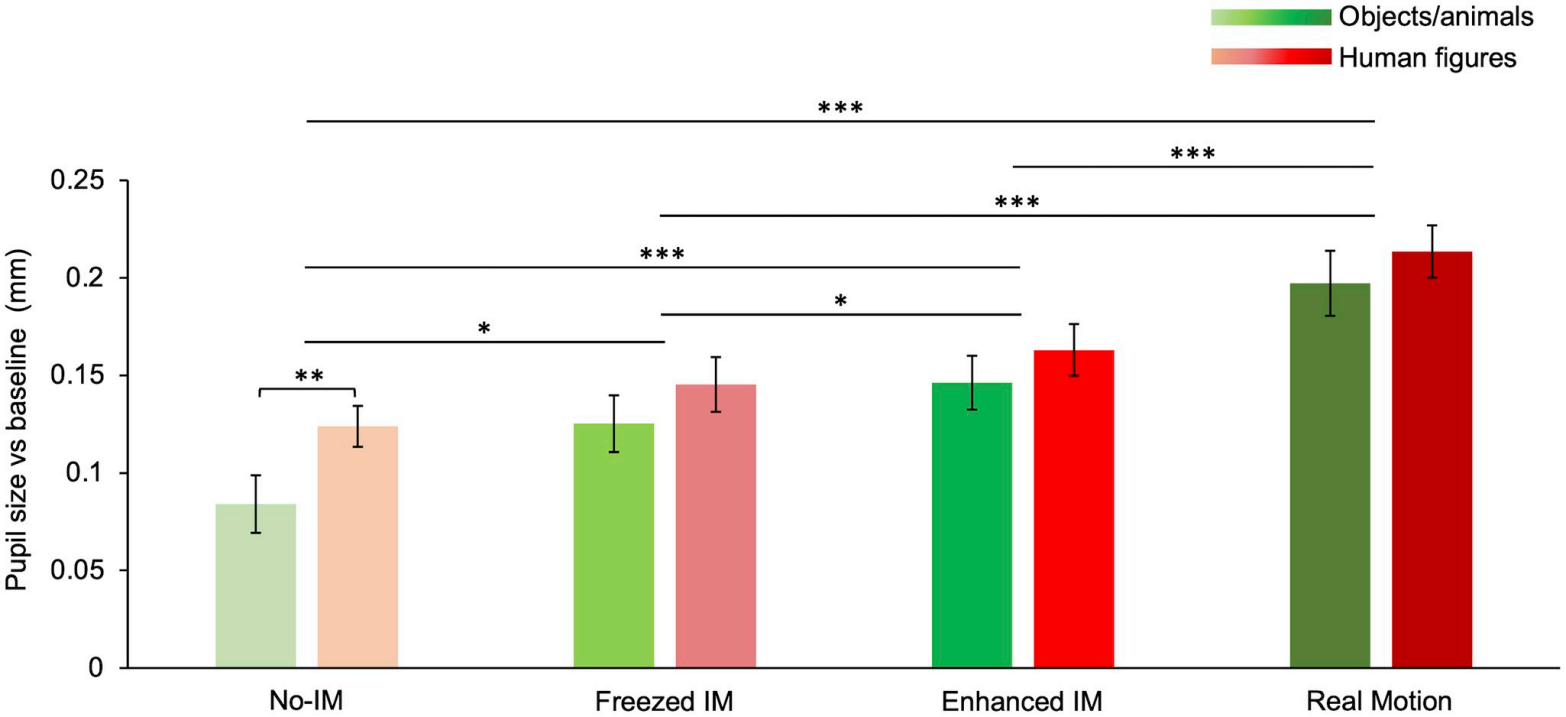
Pupillary response to different motion categories and stimulus content. Comparison between the mean pupillary dilation for different motion categories and subjects (objects/animals’: green; humans: red). Color saturation of bars increases with motion strength, in order: No-IM, *Freezed* IM, *Enhanced* IM, Real Motion (see S1 List for stimuli). Error bars are SE. Asterisks mark statistically significant post-hoc comparisons: * *p* < 0.05, ** *p* < 0.01, *** *p* < 0.001. All data shown were corrected based on each observer’s motion rating test.

Average pupil dilation in response to different motion categories divided according to stimulus content (Fig 5) shows that real motion induces the largest mean pupil dilation followed by images with *enhanced* IM and *freezed* IM, and last No-IM (the same trend observed for the pooled data, compare Fig 5 with Fig 2B). Overall, there is a significant effect of motion categories (*F*(3) = 31.07, *p* < 0.001) and stimulus content (*F*(1) = 15.94, *p* < 0.001) on pupil diameter, as found with a 2-Way ANOVA—factors: motion category (four levels: No-IM, Freezed IM, Enhanced IM, and Real motion) and image content (two levels: Objects/animals and humans). No interaction between the two factors (*F*(1) = 1.927, *p* = 0.28) is found.

Post-hoc comparisons for motion categories, independently of stimulus content, show that real motion induces more dilation than *enhanced* IM (*t*(3) = -4.54, *p* < 0.001), *freezed* IM (*t*(3) = -4.88, *p* < 0.001), and No-IM (*t*(3) = -7.77, *p* < 0.001). No-IM induces less dilation than *freezed* IM (*t*(3) = 3.23, *p* < 0.05), and *enhanced* IM (*t*(3) = 6.10, *p* < 0.001). Interestingly, the difference between *freezed* IM and *enhanced* IM is also significant (*t*(3) = 3.06, *p* < 0.05), showing that the greater the motion strength in the pictures the stronger the pupil dilation.

Multiple comparisons show that the statistical difference between human and non-human subjects is due to the response to images in the No-IM category (*t*(3) = 3.86, *p* < 0.01), although in all motion categories human subjects elicit a larger dilation than non-human (compare red bars with green bars in Fig 5).

**Fig 6:**
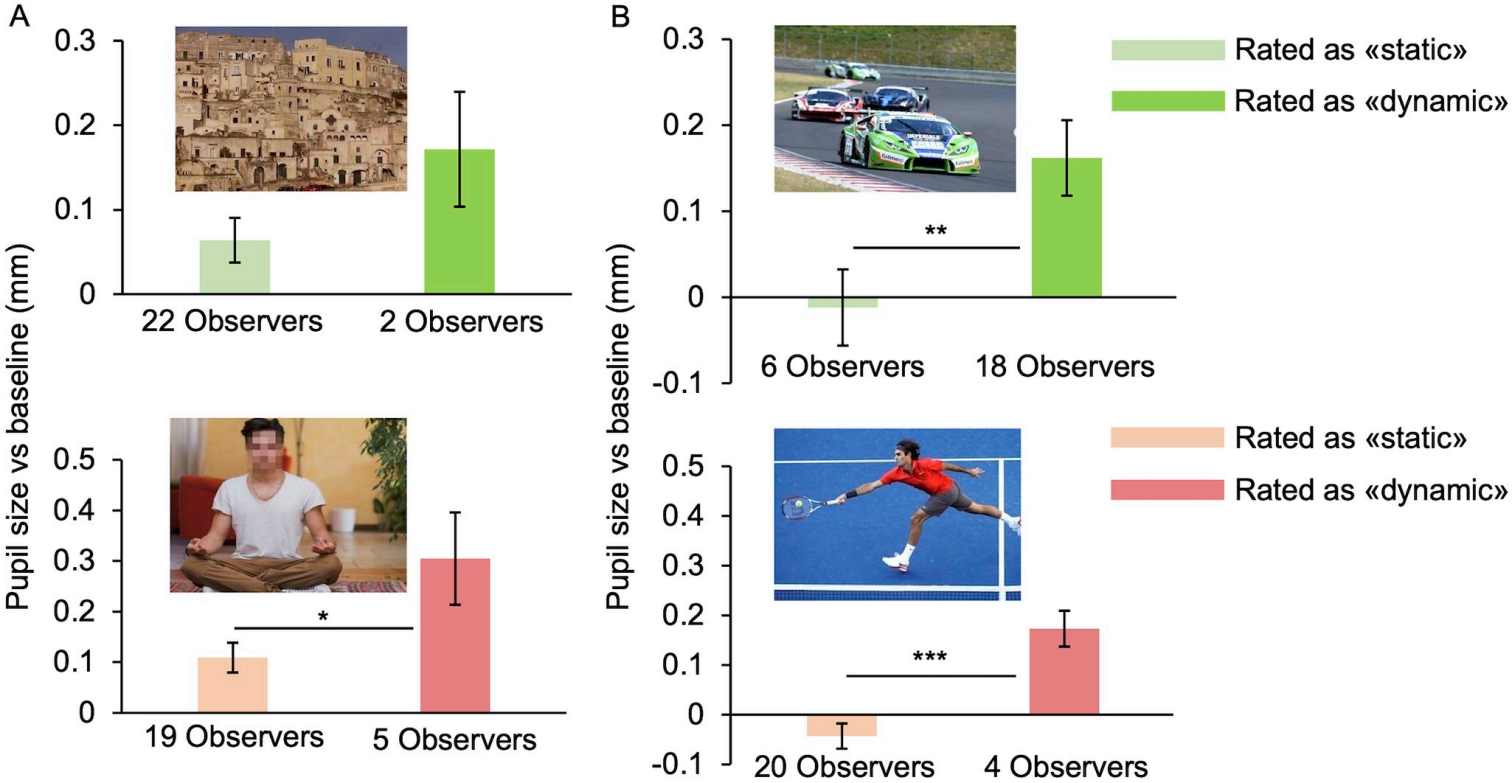
Effect of subjective interpretation of implied motion. **(A)** Photos (n° 3 and 49 of S1 List, copyright-free) belonging to No-IM category that have been rated as dynamic instead of static. **(B)** Photos (n° 20 and 55 of S1 List, copyright-free) belonging to IM category that have been rated as static instead of dynamic. Images shown in (A) and (B) are those that scored higher misinterpretation by our participants, whose number is reported under each bar. Error bars are SE. Asterisks mark statistically significant comparisons between groups, non-parametric one-tailed Mann-Whitney test, * *p* < 0.05; ** *p* < 0.01, *** *p* < 0.001.

Other interesting findings emerge when combining pupil responses with the results of the motion rating test. A weak but significant correlation emerge between subjective motion strength and pupil size (*r*(1680) = .1, p < .0001). Indeed, some photos have been categorized differently from our observers, that is, the same image was considered as “static” by some participants (answer 0 –No motion) and as “dynamic” (answer 1 –Weak motion, or 2 –Strong motion) by others. We consider a response to be a “misinterpretation” if the participant’s rating does not correspond to our nominal classification (for example if a photo of IM category has been rated as static by a participant). Examples of photos misinterpreted by more than one participant are reported in Fig 6.

The proportion of misinterpretations is not evenly distributed across categories. Considering the rating of all observers for all images, in 10% of cases, humans photos belonging to No-IM category are misinterpreted as dynamic rather than static, versus 2% of cases for objects/animals’ photos (*X*^*2*^(1) = 12.83, *p* < 0.001). In the same way, photos of human IM are misinterpreted as static only 5% of times compared with 8% for non-human IM (even if this difference is not significant; *X*^*2*^(1) = 2.21, *p* = 0.14) These results suggest that images representing human figures are more likely to be interpreted as “dynamic” compared with images with objects or animals.

Another notable result emerges from the analysis of pupillary responses to misinterpreted photos, that have not been considered in the calculation of the means of each category. Pupil size of observers that misinterpreted as dynamic the photos belonging to No-IM category, is significantly larger than pupil size of those who correctly interpreted the same images—both for human and non-human photos (examples in Fig 6A). In the same way, photos belonging to the IM category, induce smaller pupillary dilation in observers perceiving them as static than in those perceiving them as dynamic, both for human and non-human photos (examples in Fig 6B). These results do not depend on eye movements: misinterpretations and correct interpretations yield statistically compatible RMSE distributions (all pairwise KS comparisons have *p*-values > 0.05). Statistical analyses to assess the overall significance of the effect of subjective motion interpretation (see Data processing for details), yield an overall *p*-value < 0.01. Thus, pupil response seems to be modulated by the observer’s interpretation of the photos as dynamic or static, leading to larger dilation for motion.

**Fig 7:**
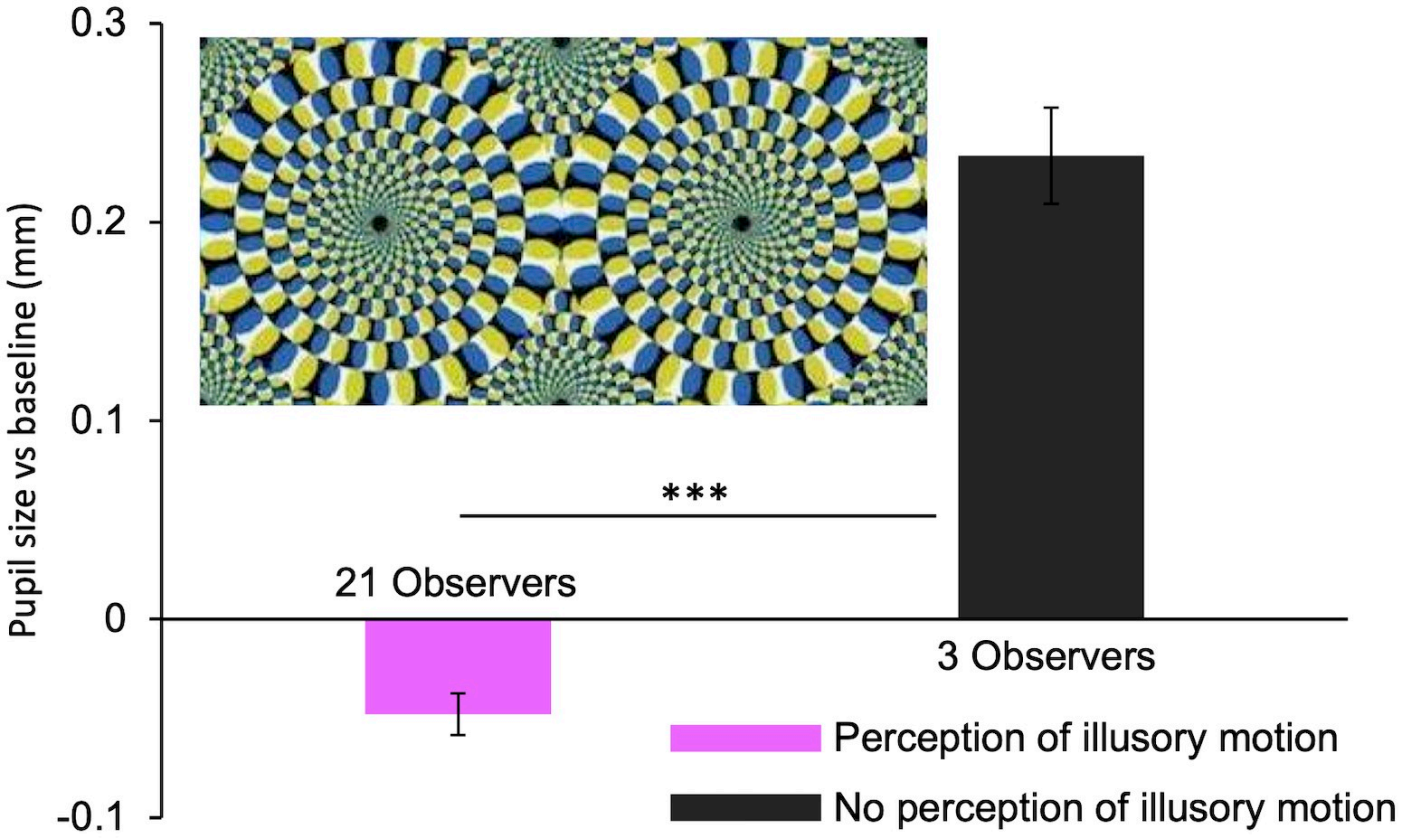
Effect of perception of illusory motion. The image shown (n° 81 of S1 List) is the one scoring the highest number of misinterpretations (3). Asterisks mark statistically significant comparisons between groups, non-parametric one-tailed Mann-Whitney test, *** *p* < 0.001.

Motion illusions also give way to a certain number of misperceptions, in which participants did not perceive any motion (image rated as "0—No Motion"). This happens in 5% of the cases, and, in each of these, the observers’ pupil size is smaller than when illusory motion is perceived. An example of this is shown in Fig 7. As for misinterpretations of photos, a non-parametric, one-tailed, Mann-Whitney ranking test was performed for all illusions that gave way to at least one misperception. Individual *p* values were combined according to Fisher’s method [99] yielding an overall *p*-value < 0.05. No difference in eye position emerges across subjects perceiving the illusions as moving versus those perceiving them as static (all RMSE distributions are statistically compatible—all pairwise KS comparisons have *p*-values > 0.05). Again, pupil size seems to be mostly dependent on the subjective perception of stimuli. Note that the perception of illusory motion induces less pupillary dilation (sometimes even constriction) whereas the lack of perception of illusory motion results in pupil dilation. This is the exact opposite of what is observed for IM.

## Discussion

In the present study, for the first time, pupil diameter of observers has been recorded while they were viewing different kinds of motion stimuli, such as movies, illusions, and photos, representing a variety of subjects. All stimuli have the same mean luminance, whereby a simple model in which pupil size exclusively depends on luminance would predict the same amount of pupillary dilation for all stimulus’ categories. Instead, pupil responses to the different motion categories result to be different, showing that there must be a pupil modulation from mechanisms other than the PLR.

Movies and photos showing figurative subjects in action (humans, objects, or animals) induce an increase in pupil size, although with differences in time courses and average dilation. Perception of real motion induces a rapid increase in pupil size after stimulus onset, reaching an average pupillary dilation greater than that produced by all other categories. Processing of implied motion also induces dilation, but with a lower increase with time and a smaller average size compared to real motion. Within IM stimuli, *Enhanced* IM, specifically created by photographers to increase our motion perception, induce more dilation than simple *freezed* IM, where subjects are photographed while they are moving. Photos of static scenes (No-IM) induce less pupil dilation than photos of dynamic subjects (IM), although sharing the same low-level features. Therefore, these results suggest that the greater the perception of movement in the stimulus, the stronger the pupillary dilation of the observer.

These findings show that the processing of real and implied motion of a variety of figurative subjects modulates the pupil diameter, an effect not investigated in previous studies, which used non-figurative stimuli [90]. The only other study measuring pupil responses to real moving stimuli used random moving dots and found that the onset of coherent motion triggers pupillary constriction [90]. This result seems to be at odds with our results on movies, but the two studies are hardly comparable. First, in Sahraie and Barbur’s experiment motion perception was generated by artificial stimuli, instead of realistic subjects in action. Realistic stimuli may activate a more complex network of cortical areas like those activated in real-life conditions. Secondly, their stimuli were shown in the periphery of the visual field while observers fixate the screen center, while our movies are directly attended in the fovea.

The observed pupillary dilation to real motion could be attributed to the activation of the sympathetic system, which regulates important bodily functions (for a review see [107]) and, once activated, causes a series of physiological reactions which prepare the organism for action, including pupillary dilation. Indeed, pupil size has been shown to be a useful indicator of physiological arousal [69–72], with direct connections to the locus coeruleus arousal network [71, 73]. Therefore, pupillary dilation in response to motion could reflect a physiological response of the system preparing for a highly dynamic situation causing arousal. Another plausible explanation may rely on the fact that pupillary dilation could be related to the need for a wider visual field to track objects in motion. We know that in real-life conditions we need a large field of view to perceive physical movement [2], and it has been suggested that increasing pupil size increases the visual field [108]. Therefore, it could be that the pupil dilates to enlarge the portion of visual field to improve the perception of objects’ movement in the periphery of the visual field. The explanations hypothesized above might be held for the effect of IM as well. That is, the same mechanisms, but to a smaller extent, might happen for photos with IM, which simulate the subject’s shift in the visual scene, also explaining the smaller pupil dilation in response to implied than real motion.

The different pupil responses to real and implied motion could also be attributed to different top-down cortical modulations of the pupil. Specifically, our findings could reflect different degrees of activation of the cortical area MT, dedicated to the processing of both real motion [8, 10, 12–15, 18, 24] and dynamic information represented in static images [26, 28, 29, 31, 44]. In the latter, MT activation increases with the strength of IM [31], then probably explaining the bigger dilation for photos showing *enhanced* IM compared to photos with *freezed* IM. The hypothesis that pupil dilation for IM may depend on the processing of the embodied motion information in the photos is also supported by the fact that, for a given photo, pupil size was bigger for those subjects that rated it as dynamic with respect to those that rated it as static.

Areas other than the prototypical motion ones could be involved in high-level cognitive processing of our stimuli, which contain complex scenes (foreground moving, detailed background, colors, etc.) with moving objects of different nature that could trigger multiple mental processes (attention, object recognition, familiarity, memory, and imagination). In fact, our data show that pupillary responses are also influenced by the type of subject represented in the stimuli, independently of its motion. Photos containing humans induce a bigger dilation than photos of non-humans of the same category. This could be dependent on the activation of additional areas in charge of visual processing of the human body and face [50–57], and/or areas specialized for human biological motion processing [16, 62–64, 109–114], and/or systems responding to an increase of the arousal due to viewing humans figures [115–117]. Also, visual scenes showing humans in action are perceived as more dynamic than representations of moving objects or animals, maybe due to our tendency to attribute intention to move to other people [58–60]. This is in agreement with the higher activation of MT during observation of human actions rather than non-biological movements [61].

A completely different and opposite effect is obtained with illusory motion. In fact, by presenting motion illusions pupil diameter decreases significantly. Pupil reaches constriction, remains constricted for a while, and finally starts to dilate slowly. The average size in this condition is therefore smaller than in all the other motion conditions.

The effect we found with motion illusions is not in line with the only other study measuring pupil response to illusory motion [89], but the inconsistency can be explained by several differences in the stimuli, experimental procedures, and data analysis used. First, Beukema and colleagues [89] measured in all trials the response to one single optical illusion, which gives way to the perception of peripheral drift, while we presented ten different illusions giving the impression of motion with different speeds and directions, making our conclusions more general. Then, more importantly, while pupil behaviours with time are almost the same in both experiments, the average diameter they obtain is much larger. They exclude from the analysis the response during the first second from stimulus onset, during which the pupil constricts, that they consider just the effect of the PLR. We argue that the effect of PRL is found during the first 350 ms after stimulus onset [86, 96], during which we measured the same amount of pupil dilation for all photos, movies, and illusions, as expected from PLR for stimuli with the same and lower luminance than the fixation slide. After 350 ms, all stimuli induced different pupil responses and, in our view, this is what makes the difference between responses to different categories.

We can only make speculations about the causes of the observed constriction for illusory motion. We could argue that the constriction does not depend on aesthetic pleasantness (not measured here), which has been found to be similar to that for symmetric non-illusory patterns [118], and generally induces pupil dilation [119, 120]. Another explanation might rely on the low-level properties of illusions, different from those of naturalistic photos, and purposely introduced to create the impression of motion [19, 21, 22]. By design, our illusions and control images have the same color saturation but induce opposite responses. Therefore, saturation does not seem to play a major role in the observed constriction. The effect might still depend on chromatic and luminance contrast [91, 121]. In any case the same illusion, therefore with the same low-level features, induce different effects in the observers according to their perception of illusory motion. This suggests that responses to illusions are not solely dependent on their low-level properties, but, at least in part, influenced by high-level motion processing.

Overall, in the same observers, perception of real and implied motion induces pupillary dilation, whereas perception of illusory motion induces pupillary constriction. These effects cannot be explained by the top-down influence of visual motion areas only. In fact, MT is activated in the same way by real, implied, and illusory motion [23–25]. It can be explained neither by a delayed response of MT+ to illusory motion, which could be the cause of the observed different time courses of pupil size, because the delay for illusory and real motion is similar [24]. Our data also exclude the possibility that pupil responses for the different types of motion are influenced by observers’ eye movement. We argue that, beside motion perception, other cognitive factors may be involved in the observed pupil differences, that reflects on top-down modulation by a larger cortical network. For example, attention may play an important role in pupil modulation. In fact, to sustain the sensation of motion, observers had to attend more closely to illusions than to real motion, leading to the speculation that other non-visual areas, part of an attentional network, are activated during observation of motion illusions, but not during the presentation of real moving objects [25]. Another plausible factor involved in the effects observed here may be a different amount of arousal triggered by artificial and naturalistic stimuli, hence a different amount of activation of the sympathetic system. The norepinephrine (NE) released from the locus coeruleus (LC) during cognitive and emotional processes, including attention and arousal, has been shown to play a critical role in the pupil dilation [71, 122–125]. Thus, the pupillary modulation observed here might also be mediated, at least in part, by the activation of the LC. Also, perception of real motion or intention to move, being fundamental for survival, has led to fine-tuned dedicated brain mechanisms in all species [5–7, 12–14, 16, 125, 126], while artificial motion illusions may rely only in part on such specialized motion mechanisms. Another plausible explanation may be the intrinsic difference between realistic photos/movies and artificial illusions reflecting on differences in the low-level visual features of these stimuli, as discussed above.

Finally, all our data, independently on the type of motion, show a strong relationship between the pupil diameter of observers and their subjective perception and interpretation of dynamicity. This confirms previous studies finding that the subjective interpretation of complex images has a relevant effect on pupil size [81, 83, 84, 87, 88], inducing responses that, in some cases, can override the simple PLR.

## Conclusions

The present study provides further evidence that, besides the simple subcortical system mediating the PLR, the pupil is sensitive to top-down cortical modulations. We demonstrated that high-level processing of visual motion influences the pupil diameter, and the effect may be different based on the nature of motion: real, implied, or illusory. This suggest that there are distinct mechanisms for the analysis of the different types of motion, despite we are not able to identify with certainty the neural pathways underlying these top-down modulations of pupil. Also, the modulation reflects the observer’s interpretation of the images as dynamic or static. For this reason, we argue that testing the subjective interpretation of each stimulus for each participant has become a necessity in pupillometry studies when using complex and ambiguous stimuli.

## Supporting information

S1 List Web sources of all stimuli. Photos and movies are divided by type of subject (objects/animals, humans) and motion category (No-IM, *freezed* IM, *enhanced* IM, real motion). Some images have been cut with respect to the originals to better fit with the assigned nominal category.
(XLSX)
